# Novel Method to Assess Endothelial Function via Monitoring of Perfusion Response to Local Heating by Imaging Photoplethysmography

**DOI:** 10.3390/s22155727

**Published:** 2022-07-30

**Authors:** Alexei A. Kamshilin, Valeriy V. Zaytsev, Anzhelika V. Belaventseva, Natalia P. Podolyan, Maxim A. Volynsky, Anastasiia V. Sakovskaia, Roman V. Romashko, Oleg V. Mamontov

**Affiliations:** 1 Institute of Automation and Control Processes of the Far Eastern Branch of the Russian Academy of Sciences, Vladivostok 690041, Russia; zaytsevphoto@gmail.com (V.V.Z.); ange@iacp.dvo.ru (A.V.B.); podolian@iacp.dvo.ru (N.P.P.); maxim.volynsky@gmail.com (M.A.V.); sakovska86@mail.ru (A.V.S.); romashko@iacp.dvo.ru (R.V.R.); mamontoffoleg@gmail.com (O.V.M.); 2 Department of Circulation Physiology, Almazov National Medical Research Centre, Saint Petersburg 197341, Russia; 3 School of Physics and Engineering, ITMO University, Saint Petersburg 197101, Russia; 4 Institute of Therapy and Instrumental Diagnostics, Pacific State Medical University, Vladivostok 690002, Russia

**Keywords:** endothelial dysfunction, imaging photoplethysmography, local hyperthermia

## Abstract

Endothelial dysfunction is one of the most important markers of the risk of cardiovascular complications. This study is aimed to demonstrate the feasibility of imaging photoplethysmography to assess microcirculation response to local heating in order to develop a novel technology for assessing endothelial function. As a measure of vasodilation, we used the relative dynamics of the pulsatile component of the photoplethysmographic waveform, which was assessed in a large area of the outer surface of the middle third of the subject’s forearm. The perfusion response was evaluated in six healthy volunteers during a test with local skin heating up to 40–42 °C and subsequent relaxation. The proposed method is featured by accurate control of the parameters affecting the microcirculation during the prolonged study. It was found that in response to local hyperthermia, a multiple increase in the pulsation component, which has a biphasic character, was observed. The amplitude of the first phase of the perfusion reaction depends on both the initial skin temperature and the difference between the basal and heating temperatures. The proposed method allows the assessment of a reproducible perfusion increase in response to hyperthermia developed due to humoral factors associated with the endothelium, thus allowing detection of its dysfunction.

## 1. Introduction

Microcirculation plays a very important role in the trophic provision of tissues and the maintenance of tissue metabolism. The vascular endothelium performs a number of important functions in the body, maintaining the function of vessels through complex interactions with smooth muscle elements of vascular wall cells [1]. In particular, it regulates vascular tone through the release of vasoactive substances such as nitric oxide (NO), prostacyclin, endothelial hyperpolarizing factors, and vasoconstrictors. Moreover, the endothelium regulates the blood coagulation system due to the effect on the platelet and coagulative (to a lesser extent) link of hemostasis and on the fibrinolytic system [2]. It also participates in the production of cytokines that regulate the inflammatory process [3]. In any genesis of cardiovascular disease, endothelial system imbalances associated with insufficient endothelium-dependent vasodilation in response to vasoactive stimuli due to insufficient NO production are detected. The impaired production of NO, which is produced by nitric oxide synthases (NOS), is associated with endothelial dysfunction. Thus, a decrease in endothelial function has been identified in patients predisposing to atherosclerosis and cardiovascular diseases [4,5], smoking [6], dyslipidemia [7], diabetes mellitus [8], and obesity [9]. Therefore, the evaluation of vasodilating properties mainly caused by NO production can provide information about the integrity and function of the endothelium that has important prognostic value both for the detection of primary cardiovascular events and for subsequent determination of disease outcomes and treatment results.

At the initial stage of atherosclerosis formation, endothelium dysfunction is observed. Endothelial dysfunction was initially determined in epicardial coronary vessels by invasive methods, such as assessing the response to acetylcholine [10] or the coronary reserve [11]. Nevertheless, noninvasive visualization methods such as positron emission tomography were also used [12]. Given the invasive nature of the assessment of the endothelial function of the coronary arteries, as well as the high cost of positron emission tomography, methods for assessing the functions of peripheral vascular vessels are most often used in real clinical and scientific practice. There are two methods that are most commonly used for the noninvasive assessment of endothelial function in peripheral vessels: measuring the diameter of the brachial artery with high-resolution ultrasound (BAUS) [13] and by measuring the pulse wave amplitude with a finger plethysmograph (peripheral arterial tonometry, PAT) in response to reactive hyperemia [14]. However, both methods, along with a high diagnostic value, demonstrate significant drawbacks. In the BAUS method, such disadvantages are a strong dependence on the operator, which makes the results of different laboratories difficult to compare, and significant variations of parameters on different measurement days [15]. There are also significant limitations of the PAT method associated with the high cost of a study (pneumatic probes are throwaway), the sensitivity of the results to various external and internal factors, and the significant variability of the results obtained in the same subject [16].

Along with the described methods, there are alternative ways to measure vasomotor reactivity to physiological stimuli, the use of which can have decisive advantages for a more accurate quantitatively reproducible assessment of endothelial function. Laser Doppler flowmetry (LDF) is a frequently used method to noninvasively assess blood flow response to various stimuli, with a local heating test being popular among them [17,18,19,20].

The participation of skin microcirculation in human thermoregulation is characterized by the extreme lability of blood flow in response to temperature influences [21]. Local heating of the skin causes local hyperemia that is characterized by a biphasic increase in cutaneous blood flow: a rapid initial peak is observed within 2–5 min after the start of heating, followed by a brief nadir and then by a prolonged plateau after 20–30 min of warming [17,21]. The initial rapid phase is mainly caused by the axon reflex of local sensory nerves [22]. It was shown that the cation-selective ion channel of vanilloid type 1 (TRPV1), mainly localized on sensory nerves, makes an important contribution to the axon reflex [23].

The increase in perfusion in the second phase originates from the humoral response mainly related to endothelial factors, among which NO accounts for about two-thirds of the contribution [22]. Confirmation of the role of the endothelium was obtained by Kellogg et al. [24], who found that the endothelial inhibitor NOS does not significantly affect the reflex vasodilator response but reduces the increase in cutaneous blood flow by more than 50% with local heating. These data convincingly demonstrate the important role of NOS in the cutaneous vasodilator reaction in response to local skin heating. Adenosine and TRPV1 not only increase NO production but also have an effect independent of NO on cutaneous blood flow [22]. The endothelial hyperpolarizing factor, part of the action of which depends on epoxyeicosatrienoic acid, is also involved in the formation of the second phase of the blood flow response to local heating [25]. Combined blockade of the NO pathways and endothelial hyperpolarizing factor almost completely suppresses plateau hyperemia with a synergistic effect, suggesting mutual activation between these two pathways [25]. Evidently, cutaneous thermal hyperemia can be used to determine signs of globalized microvascular dysfunction [21,26].

Most of the features of the microcirculation response to local skin heating were detected using the LDF method. However, the main disadvantage of this method is the low reproducibility of the results and the dependence of the sensor readings on the optical properties of the skin. Moreover, most LDF probes assess blood-flow parameters at a single point in contact with the skin, whereas the probe’s pressure on the skin is not controlled. Some researchers associate the low reproducibility of the LDF method with variations in the site of measurements [19]. It is worth noting that despite almost half a century of history of using the LDF method in microcirculation research, it is still not used in clinical practice. Recently, Lapitan and Rogatkin suggested that the reason for LDF drawbacks is more fundamental and related to the nature of coherent light used in this technique [27]. To improve the performance of an optical technique in a blood flow assessment, they proposed the use of incoherent light, as is commonly used in the photoplethysmography (PPG) method. However, their particular prototype is a typical PPG-probe that operates in contact with the skin and evaluates the blood-flow parameters at a single point. In contrast, the method of imaging PPG (iPPG) has recently become increasingly used to estimate tissue perfusion in vivo, allowing for the quantification of the spatial distribution of the blood flow parameters [28,29]. Moreover, it was recently shown that iPPG allows for the assessment of the local distribution of arterial vascular tone [30], as well as vascular resistance [31].

The aim of the present study is to demonstrate the feasibility of the iPPG method to evaluate the microcirculation response of the forearm skin to moderate local heating in order to develop new technology for assessing endothelial function.

## 2. Materials and Methods

### 2.1. Participants and Study Protocol

Six healthy adult men aged 38–52 years were recruited for this study. The following conditions constituted exclusion criteria: (i) presence of cardiovascular disease risk factor such as cigarette smoking, hyperlipidemia, diabetes, and hypertension, (ii) patients with severe concomitant systemic diseases, including respiratory, hepatic and renal failure, and skin diseases, (iii) patients with a history of drug dependence or persistent alcohol consumption, which may adversely affect the patient’s compliance with the performance of the study procedures. Before the test, every potential participant underwent a preliminary screening examination, which consisted of the collection of complaints and anamnesis, and a general clinical investigation, including an objective examination, electrocardiography, and blood tests for glucose, creatinine, and cholesterol. The subjects who were found to have at least one of the above exclusion criteria were not allowed to take the test. An individual participant registration card was filled out for each subject admitted to the test. The anthropometric characteristics of the subjects are presented in Table 1.

All of the tests were performed in a dark laboratory room at the Institute of Automation and Control Processes. The room temperature was maintained at 23 ± 1 °C. After fifteen minutes of acclimatizing to the laboratory environment, the subject was asked to sit comfortably on a chair in a relaxed posture, leaning back on the chair with legs uncrossed and normal breathing. His right hand was conveniently positioned on a table at heart level. The heating element was located on the lower third of the forearm outside. After installing the heating element, we waited another 10 min to balance the temperatures and normalize the blood flow before starting the measurements.

The perfusion response to local heating of the subject’s forearm area was assessed in three stages. First, a video of the examined area was continuously and synchronously recorded with both an electrocardiogram (ECG) and skin temperature for five minutes. Second, the forearm’s area was heated up to 40–42 °C at a heating rate of about 3.5 °C per minute to avoid painful sensations [32]. The achieved level of skin temperature was maintained for several minutes. The perfusion response for each of the six subjects was assessed three times with different heating durations of about 7, 13, and 19 min. No more than one study of each subject per day was carried out. Then, in the third stage, the heater was turned off, and the skin temperature relaxed naturally. At all stages of the local heating test, the temperature, video, and ECG were continuously recorded.

### 2.2. Measuring System

**Figure 1:**
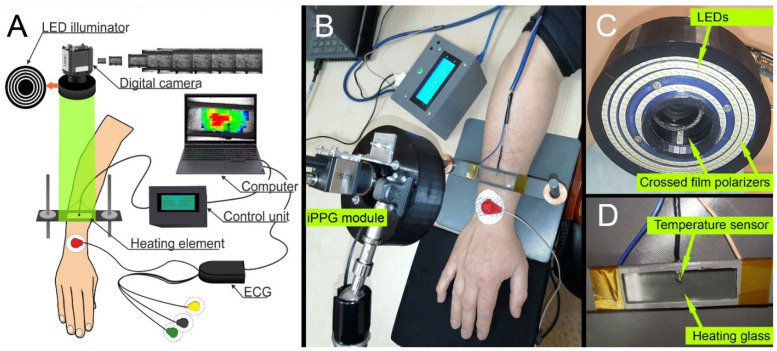
Schematic and photograph of the multimodal system for assessing blood perfusion during the local heating. (**A**) Layout of the multimodal system consisted of three computer-controlled modules (iPPG, heating, and ECG). (**B**) Photograph of the multimodal system. (**C**) IPPG module including digital monochrome camera with the lens. (**D**) Heating element with transparent conductive glass framed in the plastic and built-in temperature sensor.

To implement the above study protocol, we used a custom-made multimodal system that includes an iPPG module, an electronically controlled skin-heating module, an electrocardiograph, and a personal computer to control all components of the system and process experimental data. A schematic of the system is shown in Figure 1A. The iPPG module consists of a digital monochrome camera UI-3060CP-M-GL (Imaging Development Systems GmbH, Obersulm, Germany) with an M1214-MP2 lens (Computar, Tokyo, Japan) having a variable adjustable aperture of f = 1.4–16, minimum focusing distance of 18 cm, a viewing angle of 89° × 67°, and the ability to smooth manual focus adjustment (Figure 1B). The camera with the lens was fixed in a specially designed housing along with a light-emitting diode (LED) illuminator shown in Figure 1C. The LEDs were situated in concentric rings around the lens and emitted at the wavelength of 530 ± 25 nm. This geometry of the iPPG module provided fairly uniform illumination of the subject’s skin with the angles of illumination and observation close to the normal to minimize the ballistocardiographic artifacts [33]. All of the LEDs were covered by linear polarizing films (Edmund Optics, 0.18 mm thickness). A similar polarizing film but with an orthogonal orientation of the transmission axis covered the lens. The extinction ratio of the polarization filter was 9000:1. Such filtration reduces the skin specular reflections and motion artifacts influence on the detected PPG waveform [34]. The iPPG module was mounted on the MANFROTTO 244N Variable Friction Arm hinge-holder for easy alignment.

The heating module (Figure 1D) includes a heating element with a built-in temperature sensor and a custom-made control unit. As a heating element, we used a glass plate measuring 70 × 20 × 2 mm^3^ in a plastic frame. One side of the plate was coated with a transparent layer of indium tin oxide (ITO), having a resistance of 20 Ohm. The heating of the plate was carried out by the control unit supplying it with an electric voltage of 6 V by means of the ATmega8 microcontroller and a MOSFET transistor. The conductive layer was not in contact with the skin. To ensure thermal conductivity between the glass plate and the skin, liquid petrolatum (Vaseline oil, Flora Kavkaza, Karachay-Cherkess Republic, Russia) was applied. The frame with the heating element (total weight of 100 g) slightly pressed the subject’s forearm, as seen in Figure 1B, to provide stable thermal contact. The border of the liquid petrolatum was readily discerned in the recorded video frames, thus allowing us to assess the area of the skin–glass contact and estimate the pressure exerted on the skin. The external pressure on the skin was 12.9 ± 1.4 mmHg for all of the subjects under study.

The skin temperature was monitored by using a K-type thermocouple (chromel-alumel) located between the glass plate and the subject’s skin, as shown in Figure 1D. Since the thermocouple is within the liquid petrolatum, it adequately measures the temperature of the skin. The control unit provided temperature readings with a frequency of 5 Hz, and the data were transferred to the personal computer through a serial port. The microcontroller was commanded by software implemented on the MATLAB^®^ platform. The operator set the start and end times of heating, as well as the temperature that was maintained during the heating period. At the start point, the electric power was applied to the ITO layer providing skin heating up to the temperature of about 41 °C for two minutes. As soon as the skin temperature reached the value set by the operator, the heating of the glass plate was turned off but switched on after the one-degree temperature drop. At the endpoint of heating, the power supply of the ITO layer was completely stopped. In addition, to ensure the subject’s safety, both the instant skin temperature and the current stage of the heat test were displayed on the liquid crystal display of the control unit.

To record ECG, we used a digital electrocardiograph (model Kardiotekhnika-EKG-8, Incart Ltd., St. Petersburg, Russia) equipped with an analog–digital converter operating at the sampling rate of 1000 Hz. One of the converter’s channels was used to record sync pulses from the video camera. The ECG module transfers the data to the personal computer via the USB 2.0 port. Therefore, the recorded frames were synchronized with ECG with an accuracy of 1 ms.

The iPPG module was adjusted in such a way that the maximum area of the frame image was occupied by the heating element in the forearm. The frame exposure was adjusted individually for each subject considering his skin color in order to achieve the maximal response of the camera sensor but to avoid overexposed pixels in the skin area under study. The videos were recorded at 36 frames per second with a resolution of 376 × 256 pixels in an 8-bit format and transferred into a personal computer via the USB 3.0 port. In the computer, the frames were saved frame-by-frame on the solid-state hard disk in the PNG format.

### 2.3. Data Processing

All of the recorded data were processed offline by custom software also implemented in MATLAB^®^ (Version R2020a, The MathWorks, Inc., Natick, MA, USA, 2020). In the first stage, to minimize the impact of motion artifacts, we applied a digital image stabilization algorithm developed recently by our group [35]. Since different skin regions can shift in different directions, the entire frame of the image was divided into 64 segments measuring 32 × 47 pixels with subsequent compensation for the displacement of each segment independently.

In the second stage, an image area inside the visible boundary of the liquid petrolatum was selected. Further perfusion evaluation was carried out in this selected area. The image was divided into small regions of interest (ROI), sizing 15 × 15 pixels, which is about 2 × 2 mm^2^ in the forearm plane. The adjacent ROIs shared a common border without overlapping. A PPG waveform was calculated as a frame-by-frame evolution of the mean pixel value in every ROI. As is known, a raw PPG waveform consists of an alternating component (AC) modulated at the heartbeat rate and a slowly varying component (DC) [36]. Since both components are proportional to the incident light intensity, possible illumination unevenness was compensated by calculating the AC/DC ratio. At this stage, the normalized waveforms (AC/DC) in all selected ROIs for the entire duration of the test (about 60 min) were computed.

In the third stage, we assessed the spatial distribution of the amplitude of the pulsatile component (APC) at several time points. To this end, the normalized PPG waveform with duration a of 15 cardiac cycles was selected at the chosen time point. Then, it was cut into individual PPG pulses so that the beginning of each pulse coincided with the corresponding R-peak of the ECG. The mean PPG pulse was calculated as an ensemble-averaged 15 pulses [30,35]. Synchronously recorded ECG served as a cardiac timing reference to ensure a physiologically correct assessment of the blood–pulse wave. APC was defined as the difference between the maximum and minimum values of the mean PPG pulse. The parameter APC was estimated in each pixel, thus allowing us to calculate two-dimensional maps of the distribution of APC over the area of local heating.

**Figure 2:**
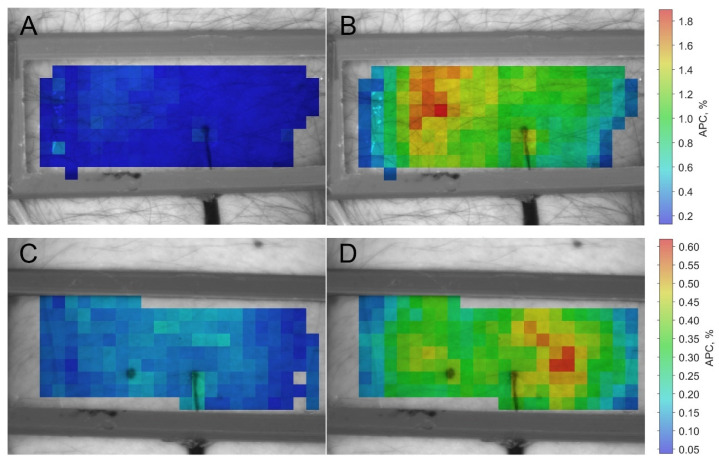
Spatial distribution of the APC parameter (perfusion index) in the region of local heating for two representative subjects. Images (**A**,**B**) with superimposed maps refer to one subject, while (**C**,**D**) to another. APC maps in panels (**A**,**C**) were assessed in the baseline, whereas maps in panels (**B**,**D**) were evaluated 200 s after the heating element was switched on. The color bars on the right show APC percentagewise, and the scales for basal perfusion and at the time of heating are the same but differ in absolute value for each subject.

**Figure 3:**
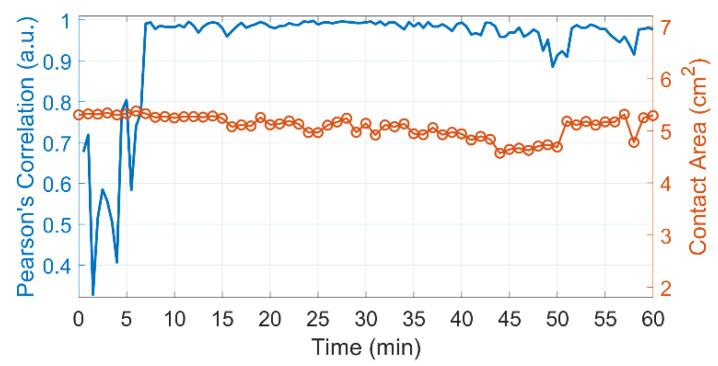
A typical example of the dynamics of experimental parameters for one of the subjects. The blue curve shows the Pearson correlation coefficient between the spatial distributions of perfusion estimated every half minute. The red curve shows the area of contact between the heating element and the skin. The heating element was turned on at the 5th and off at the 25th minute.

Examples of APC mapping overlaid on the monochrome images of the area under study are shown in Figure 2 for two subjects. Here, panels (A) and (B) refer to one subject, while panels (C) and (D) refer to another. The maps of panels (A) and (C) in Figure 2 were assessed in the baseline, 20 s before the heating element was turned on, whereas the panels (B) and (D) show APC distribution 200 s after the heating began. It was shown that the APC parameter is a measure of tissue perfusion [30,37,38] and can be referred to as the perfusion index. It is encoded in pseudo colors with a scale shown on the right: red for greater perfusion and blue for smaller. Note that the scales are the same for the baseline and hot mappings but different for different subjects. As seen, the local heating results in a strong increase in the perfusion for both subjects. However, the perfusion index is unevenly distributed over the area being heated. Comparing the APC distributions of different subjects (Figure 2B vs. Figure 2D), one can see that the areas with the greatest increase in perfusion caused by heating are located in different parts of the heating element. Despite the differences in the APC distributions of different subjects, in the course of every heating test, the perfusion distribution remained unchanged after reaching the maximal skin temperature, as illustrated by the high Pearson correlation coefficient (*r* > 0.95, *p* < 0.001) between the APC maps assessed every half minute (see Figure 3, blue curve). The presence of an identifiable petrolatum border on recorded images allowed monitoring of changes in the contact area of the heating element with the skin. It was found that the contact area varies in time, as seen in Figure 3, red curve. The contact area change was about 15–20 percent during the 60-min test, which was probably due to the subject’s arm motion.

At the fourth stage of the algorithm, we estimated the dynamics of the mean perfusion during the local heating test (which is the main goal of our study), taking into account the observed features of the perfusion distribution. To this end, we first determined the coordinates of the ROIs with a high perfusion index on the APC map assessed at the moment when the maximum skin temperature was reached (7th minute), taking into account only 50% of the total number of ROIs. Thereafter, the mean perfusion of the examined area of the skin was determined as the average value for the selected half of the ROIs throughout the test, including baseline, heating, and relaxation. In other words, we estimated the perfusion index at half of the contact area characterized by a higher heating response. Here, the perfusion index APC in each selected ROI was assessed in every cardiac cycle using R-peaks of ECG as the cardiac timing reference. Therefore, the temporal resolution of the estimated perfusion dynamics was about one second.

**Figure 4:**
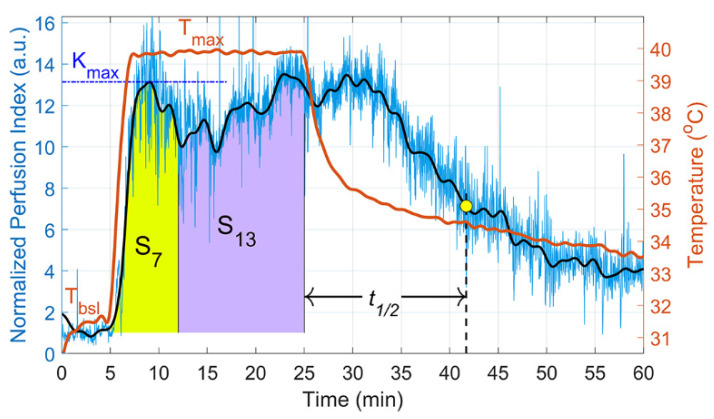
Dynamics of the normalized perfusion index for the same subject as in Figure 3. Blue curve shows unfiltered data, black curve—the same data after removing high-frequency noise, and red curve shows changes in the skin temperature.

At the final stage, for the convenience of comparing the results in different subjects, we divided the calculated mean perfusion index in each time point by the time-average perfusion value during baseline. This normalized perfusion index emphasizes the response of the vascular system to local heating in relation to the variable baseline conditions of different subjects. A typical example of the reaction of normalized perfusion to local heating is shown in Figure 4 by the blue curve. Although this raw curve is quite noisy, it clearly demonstrates a sharp increase in perfusion index in response to heating and its subsequent evolution. To reveal slowly varying perfusion changes specific to endothelial function, we filtered the data obtained by means of a low-pass filter (0.2 Hz), which was implemented using the filtfilt function in MATLAB^®^ to perform zero-phase digital filtering of the waveforms. The waveform of the filtered and normalized perfusion index is shown in Figure 4 by the black curve.

### 2.4. Statistical Analysis

The study used methods of parametric and non-parametric statistics. To test the hypothesis of the reliability of differences in dependent samples (variables), the sign test and the Wilcoxon criterion were used. Since all of the data samples for the 18 local heating tests have a normal distribution according to the Kolmogorov–Smirnov test, we used the parametric linear Pearson correlation coefficient for correlation analysis. A 95% confidence interval was used in the scatter plots. The level of significance of all of the statistical indicators is *p* < 0.05, unless otherwise indicated. The data are expressed as mean ± standard deviation (SD). Statistical analysis of the data was also performed in the MATLAB^®^ software.

## 3. Results

A multiple increase in the amplitude of skin blood flow pulsations after local heating of the outer part of the forearm was revealed in all subjects using the proposed iPPG system. Figure 4 shows a two-phase response of the cutaneous blood flow on the local heating up to 40 °C measured in subject E.

### 3.1. Dependence on the Heat-Exposure Duration

As one can see in Figure 4, the increase in skin temperature is almost immediately accompanied by a multifold increase in the APC parameter that represents tissue perfusion. These perfusion dynamics look similar to a typical dependence of the reaction of skin blood flow to local heating frequently measured by the LDF method. It was shown by numerous authors that local heating evokes an initial vasodilation response that peaks in a few minutes, followed by a brief nadir, and then secondary vasodilation to either a second extreme or plateau that can be sustained [17,39,40].

For a quantitative comparison of the perfusion dynamics, the following parameters were introduced:

- Maximum of the relative perfusion ($K_{max}$) during the first five minutes after the beginning of the heating, which indicates the perfusion enhancement;
- Initial skin temperature ($T_{bsl}$);
- Difference in the skin temperature between the heating plateau and initial temperature ($\Delta T=T_{max}-T_{bsl}$);
- Integral sum of the perfusion enhancement from the beginning of heating to the seventh minute ($S_{7}$). This parameter is shown in Figure 4 as an area shaded with yellow;
- Integral sum of the perfusion enhancement from the 7th to the 20th minute of heating ($S_{13}$), which is shown by an area shaded with lilac in Figure 4;
- Half-decay time of the perfusion index ($t_{1/2}$), which indicates the rate of decrease in perfusion after the heating is turned off. The definition of $t_{1/2}$ is illustrated in Figure 4.

**Figure 5:**
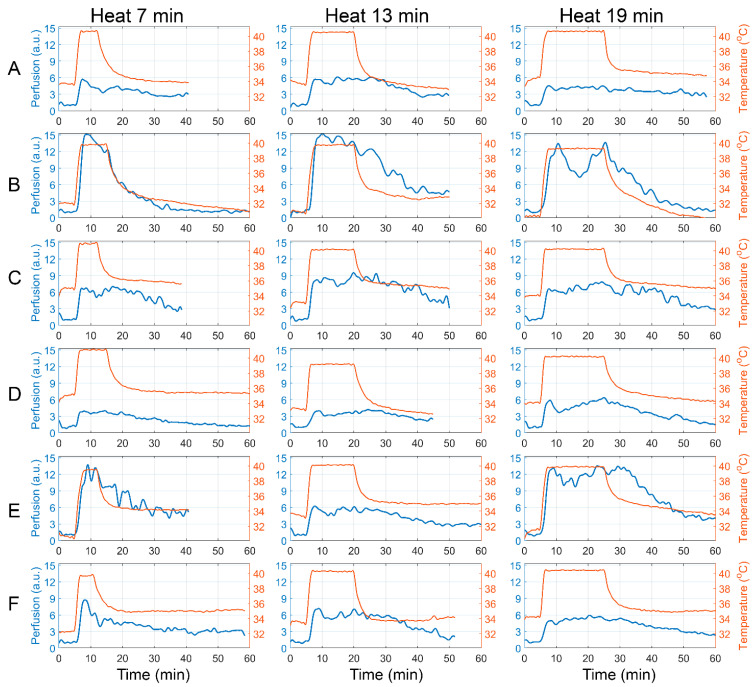
Dynamics of perfusion changes due to subjects’ forearm local heating of different duration. Rows (**A**–**F**) correspond to different subjects; the columns correspond to the different heating duration indicated in the header of each column. Blue curves show changes in the perfusion index, whereas red curves show changes in local skin temperature. The scales for the perfusion index (as well as for the skin temperature) are the same for all 18 tests. The different duration of local heating is due to the stabilization of indicators after the end of the temperature exposure and individual characteristics of each subject, controlled during the test by a medical specialist.

Figure 5 shows the responses of the perfusion in each of the six subjects on the local heating in 18 tests with three different durations of heat exposure. In this pilot study, we tried to find out which parameters of the system stronger affect the perfusion response, thus requiring precise control to increase the repeatability of the measurements. To provide a visual comparison of the responses in different tests, the scales for the perfusion index were set the same for all 18 graphs in Figure 5. Similarly, the scales for the temperature were also set the same.

As seen, the amplitude of the first response peak, $K_{max}$, varies from one subject to another in the range of 4–15 a.u. Nevertheless, $K_{max}$ in response to different heat exposures for each individual is more or less at the same level, with the exception of subject E. In this subject, one can see twofold drop in the response after 13-min of heat exposure compared to both the shorter and longer heating. The reason of such drop might be a smaller ΔT, which is due to a higher $T_{bsl}$ in this particular case. As a rule, each subject passed three tests with different durations of hyperthermia for 8–12 days. However, the difference in the time of the tests with 13- and 19-min of local heating in subject B was three months. In this exceptional case, we tried to keep both the $T_{bsl}$ and ΔT that yielded approximately the same amplitude of the initial dilator response (as seen in Figure 5B), although the difference in the carrying out of these tests was very long.

For a comparative assessment of the perfusion parameters depending on the heating time, we compared these parameters at different durations of hyperthermia. The quantitative characteristics of the skin blood-flow reaction, estimated by the proposed system, for each of the subjects with different duration of hyperthermia are presented in Table 2, Table 3 and Table 4.

**Figure 6:**
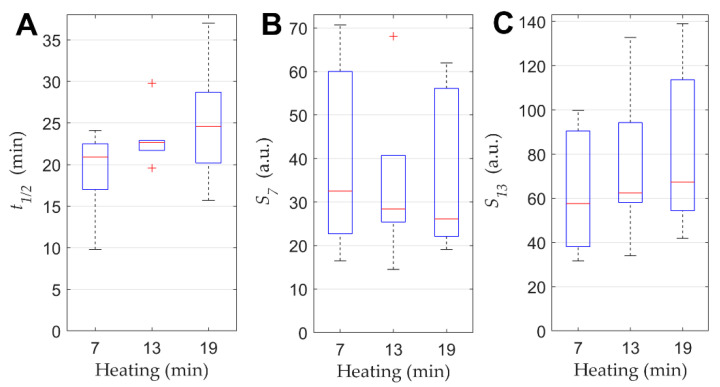
Average values of the perfusion response parameters to local heating of the skin for different heating durations. (**A**) half-decay time of the perfusion index; (**B**) integral sum of the perfusion enhancement from the beginning of heating to the 7th minute; (**C**) integral sum of the perfusion enhancement from the 7th to the 20th minute of heating. On each box, the central mark indicates the median, and the bottom and top edges of the box indicate the 25th and 75th percentiles, respectively. The whiskers extend to the most extreme data points not considered outliers, and the outliers are plotted individually using the ‘+’ symbol.

The average values of the perfusion response parameters for each heating duration are shown in Figure 6. With an increase in the duration of hyperemia, perfusion increases in the second phase of the response to heating, which is manifested in a statistically significant increase in the integral parameter $S_{13}$ in the group with 19-min heating compared to the group of 7-min heating, *p* = 0.028 (Wilcoxon test), see the right and left boxes in Figure 6C. At the same time, in the first phase of the perfusion reaction, characterized by the integral parameter $S_{7}$, no differences were found between the groups, *p* = 1.0 (Figure 6B). This indicates the need for local heating with a duration of more than 15 min for the substantial development of the second phase of the vascular response to hyperthermia.

In addition, with increasing local heating duration, there is a progressive increase in the half-decay time. In the group of subjects with 7-min hyperthermia, the average parameter $t_{1/2}$ was 19.2 ± 5.2 min, while in the group with 19-min heating, it increased to 25.1 ± 7.7 min, *p* = 0.075 (Wilcoxon test), see the right and left boxes in Figure 6A. This indicates a more inert change in blood flow with prolonged heating, which is consistent with the idea of the humoral origin of the vascular response in the late phase of hyperthermia.

### 3.2. Effect of Initial Temperature and Temperature Difference

**Figure 7:**
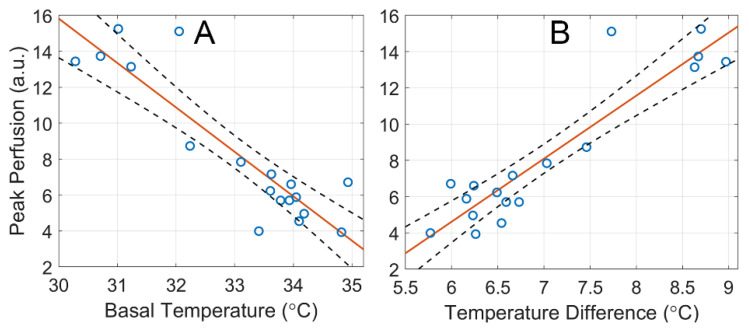
Dependence of the maximum increase ($K_{max}$) in the perfusion index during the first phase of the vascular reaction on the initial temperature $T_{bsl}$ (graph (**A**)), and on the temperature difference ΔT (graph (**B**)). Blue circles show the measured values, the red line is the best fit for the data, and black dashed curves represent a 95% confidence interval for the regression line.

Despite the fact that the temperature in the heating plateau according to the study protocol was approximately the same (about 40 °C), the degree of temperature increase differed significantly. It was mainly caused by the initial skin temperature during first five minutes of the local heating test. Correlation analysis of all 18 local heating tests revealed strong correlations between $K_{max}$ and ΔT, as well as between $K_{max}$ and $T_{bsl}$. The graph in Figure 7A shows that the amplitude of the first dilatation peak correlated negatively (*r* = −0.90, *p* < 0.001) with the initial skin temperature: the higher $T_{bsl}$, the smaller the perfusion enhancement. At the same time, $K_{max}$ positively correlates with the temperature difference ΔT: *r* = 0.92, *p* < 0.001. As one can see in Figure 7B, $K_{max}$ increases with an increase in ΔT.

From our point of view, it is the detected dependence of the amplitude of the blood flow response on ΔT and $T_{bsl}$ that causes a large standard deviation from the average values for the integral indices $S_{7}$ and $S_{13}$, as shown in Table 2, Table 3 and Table 4 and Figure 6B,C. In subsequent studies, it will be desirable to standardize both the initial skin temperature $T_{bsl}$ and the temperature difference ΔT during heating.

## 4. Discussion

Our pilot study is devoted to demonstrating a new method for assessing endothelial function based on the registration of iPPG indicators reflecting the dynamics of vascular tone in response to local skin heating. The principal difference of this method is the monitoring of a vascular reaction in a significant part of the forearm area in response to dosed local hyperthermia. The stability of local skin heating was achieved using a new multimodal system in which the heating glass contacts the skin while perfusion is assessed in a contactless way using imaging photoplethysmography.

Comparing to existing noninvasive techniques, this configuration provides a more accurate control over the parameters affecting perfusion, such as skin temperature and external pressure. It is worth noting that contact of the heating element with the skin is necessary to ensure stable thermal conductivity and thus reliable receptors response. In our system, the heating and measuring modules are separated from each other. This allowed us to design a heating element with a large area of contact with the skin, while the iPPG module can both estimate the contact area and take into account the spatial heterogeneity of the perfusion reaction to local heating. Since the iPPG signal is highly dependent on the magnitude of the external pressure on the capillary bed [41], stability of the contact pressure must be maintained in order to achieve reliability and repeatability of the results.

All studies for each of the subjects were carried out on different days. Nevertheless, the integral parameter $S_{7}$ characterizing the first phase of the vascular reaction, which is associated with the axon reflex [17], was similar in all three groups of different heating duration (see the penultimate columns in Table 2, Table 3 and Table 4), indicating the good reproducibility of the results. In addition, the good repeatability of the relative increase in perfusion in the first phase of the response at different heating duration is confirmed in Figure 5 for subjects A, B, C, and D. In contrast to the proposed multimodal system, the single-point LDF method, which is currently the most accepted technique for assessment of the cutaneous blood flow regulation during local thermal hyperemia, does not provide long-term reproducibility for measurements on the forearm [42].

At the same time, the parameter $\;S_{13}$, characterizing the second phase of the vascular reaction significantly differs in the groups with 7-min and 19-min heating (see the last column in Table 2 versus that in Table 4). The difference is obviously related to the fact that shorter heating causes a shorter duration of the humoral response associated with endothelial function. This was consistent with a tendency to slow down the recovery period of blood flow ($t_{1/2}$) as the heating duration increases (see Figure 6A). Therefore, with prolonged heating, a more pronounced and inert reaction of the blood vessels is observed. An observation should be noted indicating the dependence of the perfusion response on both the initial skin temperature and the difference in heating temperatures (see Figure 7). In order to increase the repeatability of the assessment of the perfusion reaction on the local heating, it would be desirable to maintain these parameters at the same level. The initial skin temperature reflects the systemic physiological condition of the subject, and it can vary significantly in different subjects even under the same conditions in the same laboratory. Technically, the proposed system enables to set the basal skin temperature of different subjects at the same level, which can be achieved, for example, by local preheating of the skin to 34 °C (the maximal basal skin temperature in our experiments). However, the effect of compulsory preheating on following vascular reaction to higher local heating is unknown. It requires further study before it can be recommended for use.

The principle for blood perfusion measurement by imaging photoplethysmography differs significantly from that of laser Doppler flowmetry. Whereas in the LDF method, the blood flow is assessed by measuring the Doppler shift in the frequency of the reflected light due to moving red blood cells, APC (an amplitude of the pulsatile light modulation at the heart rate) is adopted as the perfusion-related parameter in iPPG systems. The relationship between perfusion and the APC parameter is not trivial and requires special discussion. It is worth noting that our iPPG system operates under a green light, which ensures the highest signal-to-noise ratio (SNR) of a photoplethysmographic signal [43]. Even though the green light cannot interact with pulsatile arteries due to its small penetration depth (less than 0.5 mm [44]), it acquires modulation at the heart rate because of mechanical compression/decompression of the capillary bed by pulsating arteries located near the site underestimation [41]. The superficial capillary layer serves as the distributed network allowing for the quantitative characterization of the vascular tone because it primarily affects the pulsatile amplitude of nearby arteries [30]. Note that an additional increase in SNR in our multimodal system is provided by correlation processing of the video data and synchronously recorded ECG.

The proposed system features a high level of control over the parameters affecting the endothelium during prolonged measurements. Therefore, it is well suited for the assessment of low-frequency oscillations associated with endothelium activity [45].

It is worth noting that the study has several limitations. The main limitation is that it was performed in a small cohort because of the limited social interaction due to the pandemic situation (COVID-19) and the prolonged protocol of the study. Nevertheless, even with these participants, we demonstrated feasibility of applying the contactless iPPG method to evaluate the response of the vascular system to local heating during prolonged continuous measurements that are needed for the assessment of the endothelium function. Another limitation concerns the fact that we did not require any special restrictions on the intake of liquids from the study participants earlier than half an hour before the measurements, although this may affect the thermoregulation processes. Certainly, it is necessary to implement stricter restrictions on the consumption of liquids in future studies.

## 5. Conclusions

The proposed system has a number of advantages over the available methods of clinical and experimental evaluation of endothelial dysfunction. It is distinguished by its low cost, high information content, and reproducibility as an operator-independent method. However, to confirm the clinical significance of the method, additional studies are required in groups of patients with high cardiometabolic risk and with coronary pathology, as well as comparative studies with existing systems for assessing endothelial function.

## Figures and Tables

**Table 1:** Characteristics of subjects participating in the study.

Subject ID	Age, Years	Height, cm	Weight, kg	Body Mass Index, kg/m^2^
A	47	183	78	23
B	39	184	87	26
C	38	177	88	28
D	50	168	78	28
E	52	168	73	26
F	39	183	112	33

**Table 2:** Parameters of the perfusion reaction to local skin heating for 7 min.

ID	Heating (min)	ΔT (°C)	$K_{max}\;(a.u.)$	$t_{1/2}\;(\min)$	$S_{7}\;(a.u.)$	$S_{13}\;(a.u.)$
A	5.5	6.7	5.7	21.7	22.7	38.1
B	8.3	7.7	15.1	9.8	70.7	90.5
C	5.8	6.0	6.7	17.0	30.8	68.4
D	8.6	6.3	3.9	24.1	16.5	31.7
E	4.9	8.7	13.7	20.1	60.0	99.8
F	4.7	7.5	8.7	22.5	34.2	46.8
Mean ± SD		7.1 ± 0.9	9.0 ± 4.1	19.2 ± 5.2	39 ± 23	63 ± 28

**Table 3:** Parameters of the perfusion reaction to local skin heating for 13 min.

**ID**	**Heating (min)**	ΔT (°C)	$K_{max}\;(a.u.)$	$t_{1/2}\;(\min)$	$S_{7}\;(a.u.)$	$S_{13}\;(a.u.)$
A	13.5	6.6	5.7	22.9	25.4	60.4
B	12.9	8.7	15.3	19.6	68.1	158.8
C	13.8	7.0	7.8	22.4	40.7	94.3
D	14.0	5.8	4.0	22.9	14.5	34.0
E	13.5	6.5	6.2	29.8	27.6	58.1
F	13.4	6.7	6.7	21.7	29.2	64.5
Mean ± SD		6.9 ± 0.9	7.7 ± 3.6	23.2 ± 3.5	34 ± 17	78 ± 40

**Table 4:** Parameters of the perfusion reaction to local skin heating for 19 min.

**ID**	**Heating (min)**	ΔT (°C)	$K_{max}\;(a.u.)$	$t_{1/2}\;(\min)$	$S_{7}\;(a.u.)$	$S_{13}\;(a.u.)$
A	19.0	6.5	4.5	37	19.1	41.9
B	18.0	9.0	13.4	15.7	56.1	113.7
C	18.9	6.2	6.6	28.7	29.9	77.2
D	18.9	6.2	5.9	20.2	22.3	54.4
E	18.2	8.6	13.1	20.7	62.0	139.0
F	18.9	6.2	5.0	28.5	22.1	57.6
Mean ± SD		7.1 ± 0.4	8.1 ± 3.7	25.1 ± 7.7	35 ± 17	81 ± 35

## Data Availability

The data presented in this study are available on request from the corresponding author.

## References

[1] Gimbrone M.A., Garcia-Cardena G. (2016). Endothelial cell dysfunction and the pathobiology of atherosclerosis. Circ. Res..

[2] Mackman N., Tilley R.E., Key N.S. (2007). Role of the Extrinsic Pathway of Blood Coagulation in Hemostasis and Thrombosis. Arterioscler. Thromb. Vasc. Biol..

[3] Libby P., Ridker P.M., Maseri A. (2002). Inflammation and Atherosclerosis. Circulation.

[4] Cushman M., Lemaitre R.N., Kuller L.H., Psaty B.M., Macy E.M., Sharrett A.R., Tracy R.P. (1999). Fibrinolytic Activation Markers Predict Myocardial Infarction in the Elderly. Arterioscler. Thromb. Vasc. Biol..

[5] Brunner H., Cockcroft J.R., Deanfield J., Donald A., Ferrannini E., Halcox J., Kiowski W., Lüscher T.F., Mancia G., Natali A. (2005). Endothelial function and dysfunction. Part II: Association with cardiovascular risk factors and diseases. A statement by the Working Group on Endothelins and Endothelial Factors of the European Society of Hypertension. J. Hypertens..

[6] Celermajer D.S., Sorensen K.E., Georgakopoulos D., Bull C., Thomas O., Robinson J., Deanfield J.E. (1993). Cigarette smoking is associated with dose-related and potentially reversible impairment of endothelium-dependent dilation in healthy young adults. Circulation.

[7] Spieker L.E., Sudano I., Hürlimann D., Lerch P.G., Lang M.G., Binggeli C., Corti R., Ruschitzka F., Lüscher T.F., Noll G. (2002). High-Density Lipoprotein Restores Endothelial Function in Hypercholesterolemic Men. Circulation.

[8] Mäkimattila S., Virkamäki A., Groop P.-H., Cockcroft J., Utriainen T., Fagerudd J., Yki-Järvinen H. (1996). Chronic Hyperglycemia Impairs Endothelial Function and Insulin Sensitivity Via Different Mechanisms in Insulin-Dependent Diabetes Mellitus. Circulation.

[9] Steinberg H.O., Chaker H., Leaming R., Johnson A., Brechtel G., Baron A.D. (1996). Obesity/insulin resistance is associated with endothelial dysfunction. Implications for the syndrome of insulin resistance. J. Clin. Investig..

[10] Ludmer P.L., Selwyn A.P., Shook T.L., Wayne R.R., Mudge G.H., Alexander R.W., Ganz P. (1986). Paradoxical Vasoconstriction Induced by Acetylcholine in Atherosclerotic Coronary Arteries. N. Engl. J. Med..

[11] Gibson C.M., Cannon C.P., Daley W.L., Dodge J.T., Alexander B., Marble S.J., McCabe C.H., Raymond L., Fortin T., Poole W.K. (1996). TIMI Frame Count: A Quantitative Method of Assessing Coronary Artery Flow. Circulation.

[12] Schindler T.H., Schelbert H.R., Alessandra Q., Vasken D. (2010). Cardiac PET Imaging for the Detection and Monitoring of Coronary Artery Disease and Microvascular Health. JACC Cardiovasc. Imaging.

[13] Celermajer D.S., Sorensen K.E., Gooch V.M., Spiegelhalter D.J., Miller O.I., Sullivan I.D., Lloyd J.K., Deanfield J.E. (1992). Non-invasive detection of endothelial dysfunction in children and adults at risk of atherosclerosis. Lancet.

[14] Kuvin J.T., Patel A.R., Sliney K.A., Pandian N.G., Sheffy J., Schnall R.P., Karas R.H., Udelson J.E. (2003). Assessment of peripheral vascular endothelial function with finger arterial pulse wave amplitude. Am. Heart J..

[15] Deanfield J., Donald A., Ferri C., Giannattasio C., Halcox J., Halligan S., Lerman A., Mancia G., Oliver J.J., Pessina A.C. (2005). Endothelial function and dysfunction. Part I: Methodological issues for assessment in the different vascular beds: A statement by the Working Group on Endothelin and Endothelial Factors of the European Society of Hypertension. J. Hypertens..

[16] Vizzardi E., Gavazzoni M., Della Pina P., Bonadei I., Regazzoni V., Sciatti E., Trichaki E., Raddino R., Metra M. (2014). Noninvasive Assessment of Endothelial Function: The Classic Methods and the New Peripheral Arterial Tonometry. J. Investig. Med..

[17] Minson C.T., Berry L.T., Joyner M.J. (2001). Nitric oxide and neurally mediated regulation of skin blood flow during local heating. J. Appl. Physiol..

[18] Charkoudian N., Eisenach J.H., Atkinson J.L.D., Fealey R.D., Joyner M.J. (2002). Effects of chronic sympathectomy on locally mediated cutaneous vasodilation in humans. J. Appl. Physiol..

[19] Cracowski J.-L., Minson C.T., Salvat-Melis M., Halliwill J.R. (2006). Methodological issues in the assessment of skin microvascular endothelial function in humans. Trends Pharmacol. Sci..

[20] Roberts K.A., van Gent T., Hopkins N.D., Jones H., Dawson E.A., Draijer R., Carter H.H., Atkinson C.L., Green D.J., Thijssen D.H.J. (2017). Reproducibility of four frequently used local heating protocols to assess cutaneous microvascular function. Microvasc. Res..

[21] Minson C.T. (2010). Thermal provocation to evaluate microvascular reactivity in human skin. J. Appl. Physiol..

[22] Brunt V.E., Minson C.T. (2011). Cutaneous thermal hyperemia: More than skin deep. J. Appl. Physiol..

[23] Wong B.J., Fieger S.M. (2010). Transient receptor potential vanilloid type-1 (TRPV-1) channels contribute to cutaneous thermal hyperaemia in humans. J. Physiol..

[24] Kellogg D.L., Zhao J.L., Wu Y. (2008). Endothelial nitric oxide synthase control mechanisms in the cutaneous vasculature of humans in vivo. Am. J. Physiol. Circ. Physiol..

[25] Brunt V.E., Minson C.T. (2012). KCa channels and epoxyeicosatrienoic acids: Major contributors to thermal hyperaemia in human skin. J. Physiol..

[26] Roustit M., Cracowski J.-L. (2013). Assessment of endothelial and neurovascular function in human skin microcirculation. Trends Pharmacol. Sci..

[27] Lapitan D., Rogatkin D. (2021). Optical incoherent technique for noninvasive assessment of blood flow in tissues: Theoretical model and experimental study. J. Biophotonics.

[28] Mamontov O.V., Shcherbinin A.V., Romashko R.V., Kamshilin A.A. (2020). Intraoperative imaging of cortical blood flow by camera-based photoplethysmography at green light. Appl. Sci..

[29] Kukel I., Trumpp A., Plötze K., Rost A., Zaunseder S., Matschke K.E., Rasche S. (2020). Contact-free optical assessment of changes in the chest wall perfusion after coronary artery bypass grafting by imaging photoplethysmography. Appl. Sci..

[30] Lyubashina O.A., Mamontov O.V., Volynsky M.A., Zaytsev V.V., Kamshilin A.A. (2019). Contactless assessment of cerebral autoregulation by photoplethysmographic imaging at green illumination. Front. Neurosci..

[31] Volynsky M.A., Mamontov O.V., Osipchuk A.V., Zaytsev V.V., Sokolov A.Y., Kamshilin A.A. (2022). Study of cerebrovascular reactivity to hypercapnia by imaging photoplethysmography to develop a method for intraoperative assessment of the brain functional reserve. Biomed. Opt. Express.

[32] Magerl W., Treede R.D. (1996). Heat-evoked vasodilatation in human hairy skin: Axon reflexes due to low-level activity of nociceptive afferents. J. Physiol..

[33] Moço A.V., Stuijk S., de Haan G. (2016). Ballistocardiographic artifacts in PPG imaging. IEEE Trans. Biomed. Eng..

[34] Sidorov I.S., Volynsky M.A., Kamshilin A.A. (2016). Influence of polarization filtration on the information readout from pulsating blood vessels. Biomed. Opt. Express.

[35] Kamshilin A.A., Krasnikova T.V., Volynsky M.A., Miridonov S.V., Mamontov O.V. (2018). Alterations of blood pulsations parameters in carotid basin due to body position change. Sci. Rep..

[36] Allen J. (2007). Photoplethysmography and its application in clinical physiological measurement. Physiol. Meas..

[37] Lima A.P., Bakker J. (2005). Noninvasive monitoring of peripheral perfusion. Intensive Care Med..

[38] Kyriacou P.A., Shafqat K., Pal S. (2009). Pilot investigation of photoplethysmographic signals and blood oxygen saturation values during blood pressure cuff-induced hypoperfusion. Measurement.

[39] Barcroft H., Edholm O.G. (1943). The effect of temperature on blood flow and deep temperature in the human forearm. J. Physiol..

[40] Johnson J.M., Kellogg D.L. (2010). Local thermal control of the human cutaneous circulation. J. Appl. Physiol..

[41] Kamshilin A.A., Nippolainen E., Sidorov I.S., Vasilev P.V., Erofeev N.P., Podolian N.P., Romashko R.V. (2015). A new look at the essence of the imaging photoplethysmography. Sci. Rep..

[42] Roustit M., Blaise S., Millet C., Cracowski J.-L. (2010). Reproducibility and methodological issues of skin post-occlusive and thermal hyperemia assessed by single-point laser Doppler flowmetry. Microvasc. Res..

[43] Fallow B.A., Tarumi T., Tanaka H. (2013). Influence of skin type and wavelength on light wave reflectance. J. Clin. Monit. Comput..

[44] Anderson R.R., Parrish J.A. (1981). The optics of human skin. J. Invest. Dermatol..

[45] Kvernmo H.D., Stefanovska A., Kirkebøen K.A., Kvernebo K. (1999). Oscillations in the Human Cutaneous Blood Perfusion Signal Modified by Endothelium-Dependent and Endothelium-Independent Vasodilators. Microvasc. Res..

